# Differentiation of histological calcification classifications in breast cancer using ultrashort echo time and chemical shift-encoded imaging MRI

**DOI:** 10.3389/fonc.2024.1475090

**Published:** 2024-12-17

**Authors:** Yazan Ayoub, Sai Man Cheung, Boddor Maglan, Nicholas Senn, Kwok-Shing Chan, Jiabao He

**Affiliations:** ^1^ Institute of Medical Sciences, School of Medicine, Medical Sciences and Nutrition, University of Aberdeen, Aberdeen, United Kingdom; ^2^ Newcastle Magnetic Resonance Centre, Translational and Clinical Research Institute, Faculty of Medical Sciences, Newcastle University, Newcastle, United Kingdom; ^3^ Athinoula A. Martinos Center for Biomedical Imaging, Department of Radiology, Massachusetts General Hospital, Charlestown, MA, United States; ^4^ Department of Radiology, Harvard Medical School, Boston, MA, United States

**Keywords:** invasive ductal carcinoma, ductal carcinoma *in situ*, ultrashort echo time, lipid composition, chemical shift-encoded imaging

## Abstract

**Introduction:**

Ductal carcinoma *in situ* (DCIS) accounts for 25% of newly diagnosed breast cancer cases with only 14%–53% developing into invasive ductal carcinoma (IDC), but currently overtreated due to inadequate accuracy of mammography. Subtypes of calcification, discernible from histology, has been suggested to have prognostic value in DCIS, while the lipid composition of saturated and unsaturated fatty acids may be altered in *de novo* synthesis with potential sensitivity to the difference between DCIS and IDC. We therefore set out to examine calcification using ultra short echo time (UTE) MRI and lipid composition using chemical shift-encoded imaging (CSEI), as markers for histological calcification classification, in the initial *ex vivo* step towards *in vivo* application.

**Methods:**

Twenty female patients, with mean age (range) of 57 (35–78) years, participated in the study. Intra- and peri-tumoural degree of calcification and peri-tumoural lipid composition were acquired on MRI using UTE and CSEI, respectively. *Ex vivo* imaging was conducted on the freshly excised breast tumour specimens immediately after surgery. Histopathological analysis was conducted to determine the calcification status, Nottingham Prognostic Index (NPI), and proliferative activity marker Ki-67.

**Results:**

Intra-tumoural degree of calcification in malignant classification (1.05 ± 0.13) was significantly higher (p = 0.012) against no calcification classification (0.84 ± 0.09). Peri-tumoural degree of calcification in malignant classification (1.64 ± 0.10) was significantly higher (p = 0.033) against no calcification classification (1.41 ± 0.18). Peri-tumoural MUFA in malignant classification (0.40 ± 0.01) was significantly higher (p = 0.039) against no calcification classification (0.38 ± 0.02). Ki-67 showed significant negative correlation against peri-tumoural MUFA (p = 0.043, ρ = −0.457), significant positive correlation against SFA (p = 0.008, ρ = 0.577), and significant negative correlation against PUFA (p = 0.002, ρ = −0.653).

**Conclusion:**

The intra- and peri-tumoural degree of calcification and peri-tumoural MUFA are sensitive to histological calcification classes supporting future investigation into DCIS prognosis.

## 1 Introduction

Ductal carcinoma *in situ* (DCIS), a form of breast cancer characterised by abnormal proliferation of epithelial cells confined within the basement membrane of the ducts (1), has increased from 1% to 2% in 1980 to approximately 25% of all newly diagnosed symptomatic breast cancers, as a result of early detection from mammographic screening (2). Although 14%–53% of DCIS develops into invasive ductal carcinoma (IDC) (3), a large proportion, particularly low-grade DCIS, are harmless without posing an immediate risk (1). Heightened vigilance is adopted for the treatment of low-grade DCIS, in the same manner as IDC with radiotherapy, drugs, and surgical intervention (4) leading to physical trauma, sexual dysfunction, and psychological harm (5). Identification of DCIS with low risk of developing into IDC is central to avoid overtreatment (6); however, the current radiological method of mammography suffers from high false-positive and -negative rates (7) and decreased sensitivity in dense breast (8). The reliance of mammography on spatial patterns in calcified regions increases false negatives (9, 10), with approximately 15%–25% of suspected DCIS remaining unconfirmed (11, 12). Furthermore, there is a lack of strong correlation between mammographic findings and histological results, particularly in the differentiation between benign and malignant calcifications (13). Histological confirmation, although it remains the gold standard for tumour classification, is invasive and demands significant expertise, with susceptibility to partial sampling error (10). Hence, there is an unmet clinical need for radiological methods more accurately reflecting histological findings enabling non-invasive differentiation between DCIS and invasive breast cancer.

To advance the effectiveness of histology, *ex vivo* breast tissue imaging methods have been developed with demonstrable correlation with pathologically reported calcification features (14) but have not offered compelling rationale to displace current histology processes. Raman spectroscopy on biopsy specimens can profile the chemical composition of calcification to support DCIS and invasive tumour differentiation but suffers from low tissue penetration and sensitivity of autofluorescence (15). Optical coherence tomography (OCT), allowing real-time cross-sectional high-resolution tissue imaging, has been used to visualise the tissue architecture surrounding microcalcifications in *ex vivo* DCIS lesions, but suffers from imaging depth below 2 mm (16). Non-invasive radiological approaches offering critical diagnostic information before histological analysis, on the contrary, may alter patient care pathway leading to improved treatment outcome and reduced overtreatment. Dynamic contrast-enhanced (DCE) MRI, regarded as the most accurate diagnostic radiological approach in breast cancer, has a sensitivity of 89% in estimating the spatial extent of DCIS compared to 55% for mammography (17), but the image contrast reflects perfusion arising from the interplay amongst angiogenesis, vascular permeability, and vascularity (18). Vascular permeability, from time-resolved DCE MRI coupled with pharmacokinetic model, has been shown to better characterise DCIS compared against qualitative DCE MRI (19, 20), but suffers from low reproducibility owing to the susceptibility of deconvolution algorithms to biological noise (21). Shear wave elastography from ultrasound, providing a quantitative measure of tumour stiffness and in turn the presence of fibrosis, has been shown to correlate with the grades of DCIS (22, 23), but suffers from high interoperator variability and low sensitivity (23).

Calcification in the form of hydroxyapatite is associated with more aggressive DCIS (24), while spatial distribution of casting type or linear branching is associated with higher-grade DCIS (25) and increased hazard ratio in mortality (26). Elevated levels of fatty acids, particularly monounsaturated fatty acids (MUFA) and saturated fatty acids (SFA), have been associated with aggressive cancer phenotypes (27, 28), while increased fatty acid synthesis and altered lipid reserve support rapid cell proliferation and malignant transformation, hence increased risk of DCIS progression to invasive breast cancer (28, 29). Ultrashort echo time (UTE) imaging, a novel radiological method, addresses the limitation of conventional MRI to primarily soft tissue application by capturing rapid signal decay in solid state matters such as calcification (30). UTE deploys a radial scanning trajectory for data acquisition enabling minimal time lag between tissue excitation and signal detection; however, it only became adequately robust and available on clinical scanners recently (30, 31). Chemical shift-encoded imaging (CSEI) detects the evolution through time of aggregated signal from water and lipid constituents with distinctive resonance frequency with rapid field gradient switching to encode spatial information and subsequently resolves the lipid constituents using signal models with empirical constraints rather than Fourier transform to overcome incomplete signal sampling (32, 33). Calcification may be quantified using UTE targeting the rapid signal dissipation from solid matters (30, 34, 35). A difference in lipid composition has been shown in IDC compared to DCIS and normal breast tissue (27, 29), and peri-tumoural lipid composition of MUFA, SFA and polyunsaturated fatty acids (PUFA) can be quantitatively mapped using CSEI as demonstrated by us (36). We therefore set out to examine calcification using UTE MRI and lipid composition using CSEI, as markers for histological calcification classification, in the initial *ex vivo* step towards *in vivo* application.

## 2 Materials and methods

**Figure 1:**
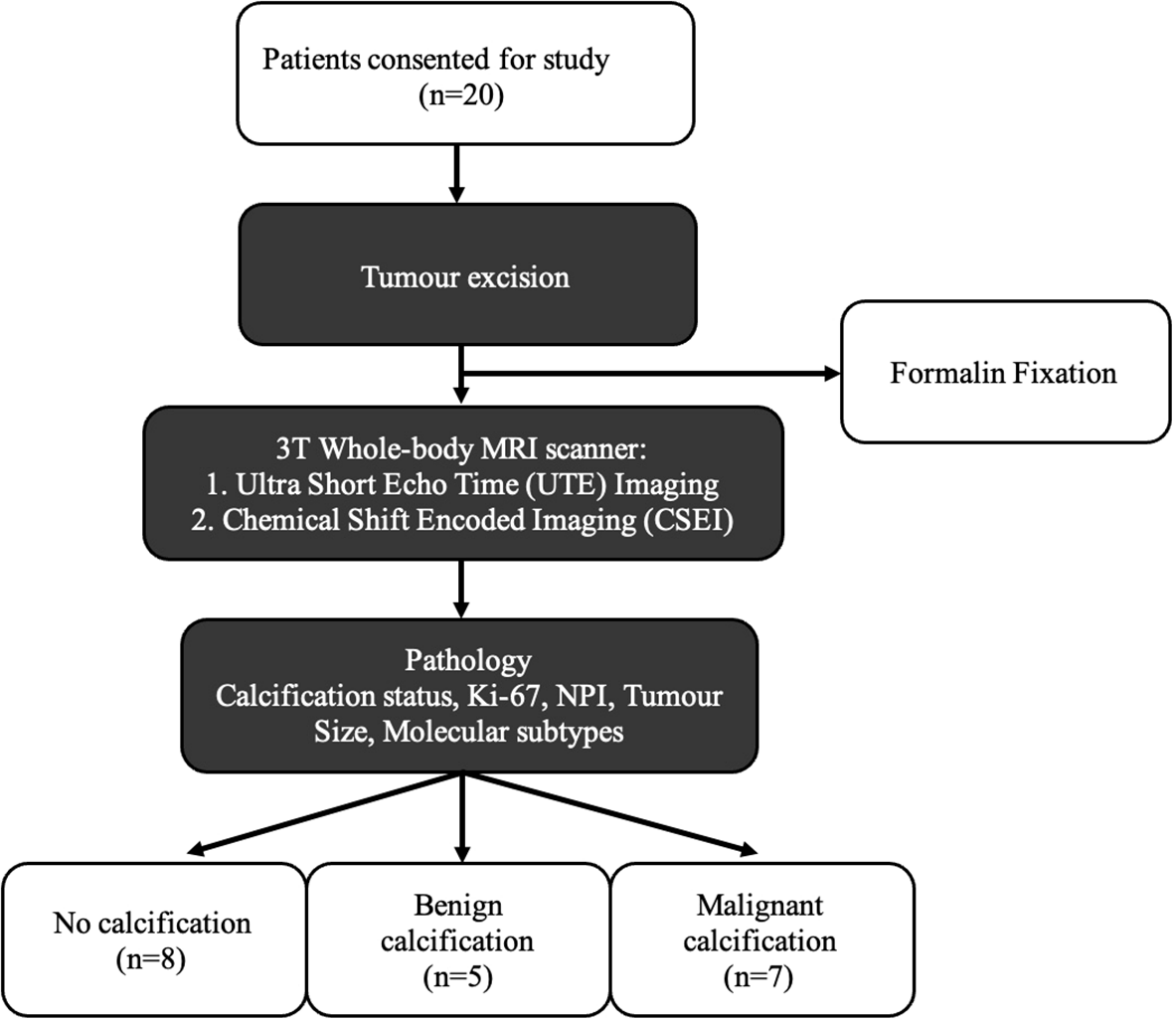
Twenty breast tumour specimens, removed from female patients undergoing wide local excision, were immediately fixed with formalin solution to prevent tissue degradation, and subsequently imaged with UTE MRI and CSEI. The degree of calcification was calculated from the UTE images and lipid composition for MUFA, SFA, and PUFA were calculated from CSEI, with histological analysis on calcification status, NPI, and Ki-67 to compare the differences between histological calcification groups.

We therefore conducted a cross-sectional study on 20 whole breast tumours freshly excised from patients to examine imaging markers against histological calcification classification. Calcification was mapped using UTE to quantify intra- and peri-tumoural calcification, and lipid composition was mapped using CSEI to quantify peri-tumoural SFA, MUFA, and PUFA (**Figure 1**). The study was approved by the North West–Greater Manchester East Research Ethics Committee (REC reference number: 16/NW/0221), and signed written informed consent was obtained from all patients prior to entry into the study.

### 2.1 Clinical procedure

Twenty whole breast tumour specimens, 10 grade II and 10 grade III, were removed from female patients, with a mean age (range) of 57 (35–78) years, with invasive ductal carcinoma undergoing wide local excision. Patients with tumour size larger than 15 mm in diameter on mammography were eligible. Patients with previous malignancies, undergoing hormonal therapy, or neoadjuvant chemotherapy were not eligible. At surgery, the freshly excised tumour specimens were placed in 10% buffered solution of formalin to prevent tissue degradation. The excised specimens were wrapped in one layer of gauze and placed at the bottom of a 1.2-L round container. A custom-designed holder, a thermoplastic ring with gauze strips was placed on top of the specimen to minimise movement, with a second and heavier ring placed on top of this first ring to provide an anchor of the whole setup inside formalin. All specimens were positioned in the isocentre of the scanner using laser cross from the scanner localisation system for precise alignment and imaged to quantify calcification and lipid composition. Routine histological analysis was performed to determine tumour grade, size, and Nottingham Prognostic Index (NPI), and research histopathological analysis on proliferative activity marker Ki-67 was conducted semi-quantitatively (36). Malignant calcification was reported in seven tumours and no calcification reported in eight tumours, with benign calcification (calcification only found in background tissue) reported in five tumours. Benign calcification was separated from no calcification to avoid potential skewed findings owing to the predisposition of tissue-level calcification.

### 2.2 Image acquisition

Image acquisition was performed on a 3T whole-body clinical MRI scanner (Achieva TX, Philips Healthcare, Best, Netherlands) using a 32-channel receiver coil for high sensitivity detection and a body coil for uniform transmission. For tumour localisation, anatomical images were acquired using clinical T_1_- and T_2_-weighted imaging sequences, with field of view of 141 × 141 mm^2^ and voxel size of 0.55 × 0.55 × 1.1 mm^3^. Calcification images were acquired using 3D-radial dual-echo UTE sequence (37), with echo times (TE) of 0.17 and 4.60 ms, repetition time (TR) of 8.5 ms, field of view of 141 × 141 mm^2^, voxel size of 2.2 × 2.2 × 2.2 mm^3^, flip angle of 5°, and 1 signal average. Lipid composition images were acquired using CSEI sequence, as detailed in our previous work (36), with an isotropic resolution of 2.2 mm, initial TE of 1.14 ms, echo spacing of 1.14 ms, 16 echoes, TR of 20 ms, flip angle of 6°, and 9 signal averages. Co-registration between the sequences was not required since higher-resolution T1- and T2-weighted anatomical images were only used for reference in the delineation of regions of interest, and UTE and CSEI images were acquired and co-localised at the same resolution in the same orientation, and hence, resampling or interpolation was not necessary.

### 2.3 Image analysis

The tumour boundary was manually delineated with reference to T_1_- and T_2_-weighted anatomical images in MATLAB (MathWorks Inc., Natick, USA) by a single operator and confirmed by a radiologist with over 10 years of experience. The intra-tumoural region was defined as the entire volume within the tumour boundary, while the peri-tumoural region was defined as a 4.4-mm rim extended outwards from the tumour boundary. A 4-mm rim for the peri-tumoural region has been shown to capture most cellular exchange between the tumour and the peri-tumoural microenvironment. A 4-mm rim contains sufficient radiomics features to support the identification of lymphovascular invasion (LVI) (38), sentinel lymph node metastasis (39), and HER-2 class and Ki-67 score (40) in invasive breast cancer. A 2.5- to 5-mm rim showed that a peri-tumoural multi texture features model of mean, entropy, skewness, kurtosis, and standard deviation may predict pathological complete response to neoadjuvant chemotherapy in DCE MRI (41). Further analysis was conducted on the differences (**Supplementary Table S1**) and correlations (**Supplementary Figure S1**) between the peri-tumoural region of 1 (2.2 mm), 2 (4.4 mm, main text), 3 (6.6 mm), and 4 (8.8 mm) voxels for MUFA, PUFA, SFA, and degree of calcification in the Supplementary Materials. There were significant correlations (*rho* > 0.6) for all metrics between threshold choices of 1, 2, 3, and 4 voxels, and there were only significant differences in MUFA (p < 0.001), PUFA (p = 0.004), SFA (p <0.001), and degree of calcification (p < 0.001) between 1 and 2 voxels.

**Figure 2:**
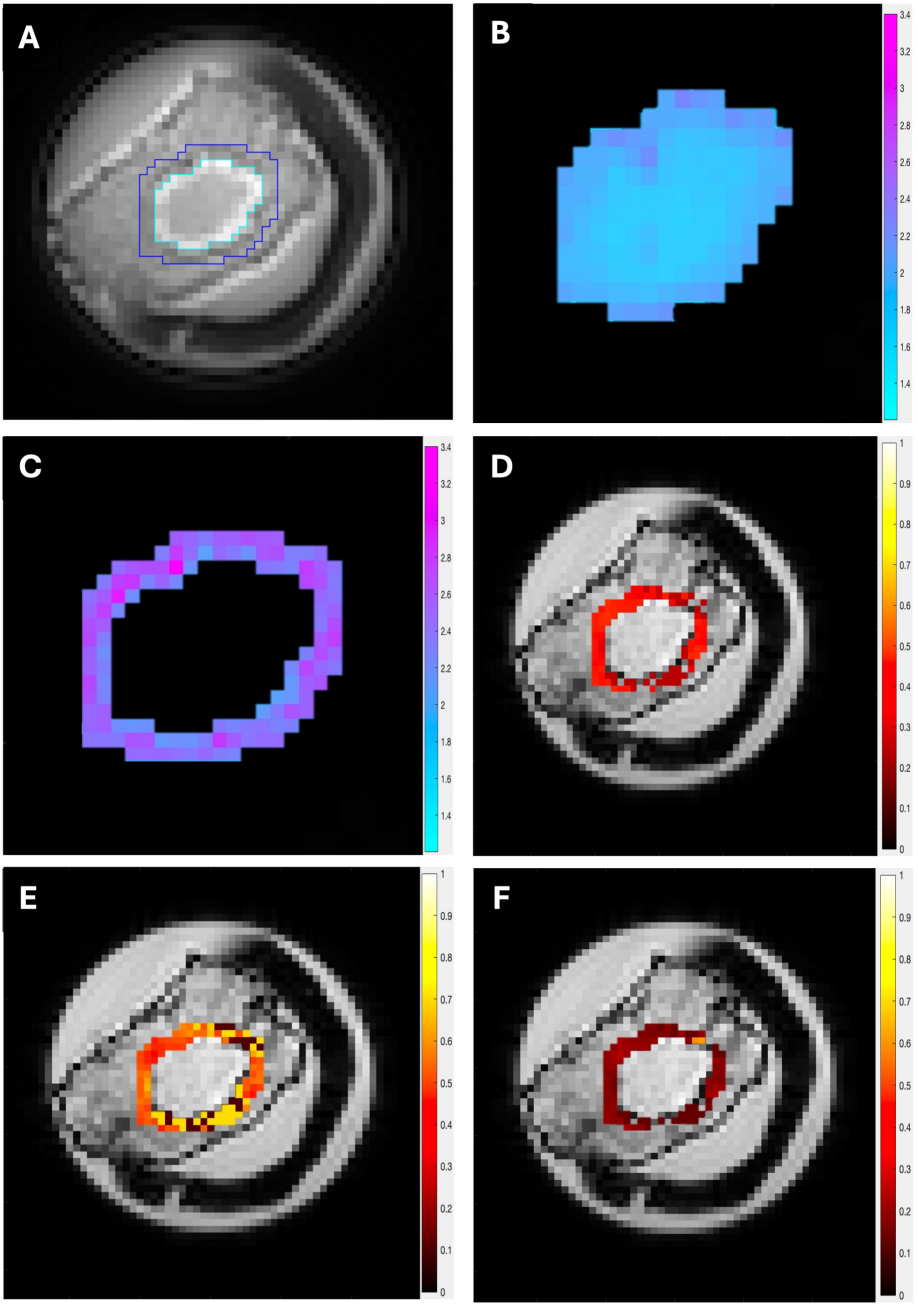
The UTE and CSEI images from a typical specimen are shown. **(A)** Delineation of the intra- (cyan) and peri- (blue) tumoural regions on UTE. The peri-tumoural region was defined as a 4.4-mm rim surrounding the tumour and delineated across the tumour volume. **(B)** Intra-tumoural degree of calcification. **(C)** Peri-tumoural degree of calcification. Peri-tumoural lipid composition maps of **(D)** MUFA, **(E)** SFA, and **(F)** PUFA overlaid on CSEI first echo image.

The intra- and peri-tumoural signals from combined solid and liquid components were quantified as the image intensity within the corresponding region from the UTE image at an echo time of 0.17 ms, while the signal from the liquid component was quantified from the UTE image at an echo time of 4.60 ms. Subsequently, the signal intensity of the two echoes from the UTE images was derived as the mean of the image intensity within the regions of interest for each echo. The degree of calcification was computed as the signal difference between the two echoes normalised by the long echo (37). The mathematical quantification approach for degree of calcification, if applied on a pixel-by-pixel basis, may significantly magnify the impact of partial volume effects and noise leading to elevated measurement error. To ensure robustness, aggregation of signal from the two echoes within the tumour and the peri-tumoural region was first computed, so that the degree of calcification reflects the ratio between the overall solid matter signal and overall liquid matter signal (42). A multi-peak spectrum model based on breast adipose tissue was used to calculate the number of double bonds in triglycerides, with specific number of double bonds in SFA, MUFA, and PUFA corresponding to the abundance of signals in the signature peaks. Subsequently, each lipid constituent was computed as a percentage of total lipid and quantified as the mean within the peri-tumoural region of interest (**Figure 2**), as detailed in our previous work (36).

### 2.4 Statistical analysis

All statistical analyses were carried out using SPSS software (Release 27.0, SPSS Inc, IL, USA), and normality was determined using the Shapiro–Wilk test. One-way ANOVA tests were performed to compare the intra- and peri-tumoural degree of calcification and peri-tumoural lipid composition amongst histological calcification groups. Tukey’s HSD *post hoc* tests were subsequently conducted to indicate the significance of differences amongst the three histological calcification groups. Spearman’s correlation tests were performed between intra- and peri-tumoural degree of calcification and peri-tumoural lipid composition against NPI and Ki-67 scores. A p-value <0.05 was considered statistically significant.

## 3 Results

There was no significant difference across patient characteristics. The patient demography is shown in **Table 1** and statistical findings in **Table 2**. The UTE MRI and CSEI MRI images from a typical specimen, with delineation of intra- and peri-tumoural regions, are shown in **Figure 2**.

**Table 1:** Patient characteristics.

Characteristics	All (n = 20)	Histological calcification			p-value
		No calcification (n = 8)	Benign calcification (n = 5)	Malignant calcification (n = 7)	
Age (years)	57 ± 14	54 ± 12	59 ± 15	60 ± 16	0.696
Tumour size (mm)	27.5 ± 7.8	28.7 ± 8.8	25.6 ± 8.7	27.3 ± 6.7	0.796
Spatial extent (mm^2^)	206.3 ± 97.7	223.0 ± 87.8	163.9 ± 90.8	217.6 ± 116.9	0.555
					
Histological grade:					
II	10	2	4	4	0.146
III	10	6	1	3	
					
Nottingham Prognostic Index (NPI)	4.44 (3.50–4.59)	4.57 (4.46 - 4.88)	3.46 (3.33 - 3.94)	4.44 (3.54 - 4.78)	0.410
Ki-67	12.85 (8.13–27.10)	18.36 (10.75 - 39.66)	11.62 (8.00 - 21.29)	10.22 (4.96 - 30.10)	0.566
Lymphovascular invasion (LVI)	12	3	3	6	0.218
Oestrogen receptor (ER+)	16	5	4	7	0.249
Human epidermal growth factor receptor 2 (HER2+)	4	3	0	1	0.387
Triple-negative breast cancer (TNBC)	3	2	1	0	0.435

**Table 2:** Degree of calcification and lipid composition in three histological calcification groups and correlation with Ki-67 and NPI scores.

	Histological calcification status			Malignant calcification vs. no calcification	Benign calcification vs. no calcification	Correlation			
	No calcification (n = 8)	Benign calcification (n = 5)	Malignant calcification (n = 7)	*p*-value	*p*-value	Ki-67		NPI	
						*p*-value	ρ	*p*-value	ρ
Intra-tumoural degree of calcification	0.84 ± 0.09	0.95 ± 0.16	1.05 ± 0.13	0.012*	0.304	0.132	−0.349	0.205	−0.296
Peri-tumoural degree of calcification	1.41 ± 0.18	1.42 ± 0.20	1.64 ± 0.10	0.033*	0.996	0.243	−0.274	0.816	0.056
Peri-tumoural SFA	0.52 ± 0.04	0.50 ± 0.02	0.48 ± 0.03	0.070	0.384	0.008*	0.577	0.448	0.180
Peri-tumoural MUFA	0.38 ± 0.02	0.39 ± 0.01	0.40 ± 0.01	0.039*	0.268	0.043*	−0.457	0.442	−0.182
Peri-tumoural PUFA	0.10 ± 0.02	0.11 ± 0.01	0.12 ± 0.02	0.168	0.588	0.002*	−0.653	0.731	−0.082

SFA, saturated fatty acids; MUFA, monounsaturated fatty acids; PUFA, polyunsaturated fatty acids; NPI, Nottingham Prognostic Index.Statistical significant differences (p < 0.05) are marked in bold and (*).

**Figure 3:**
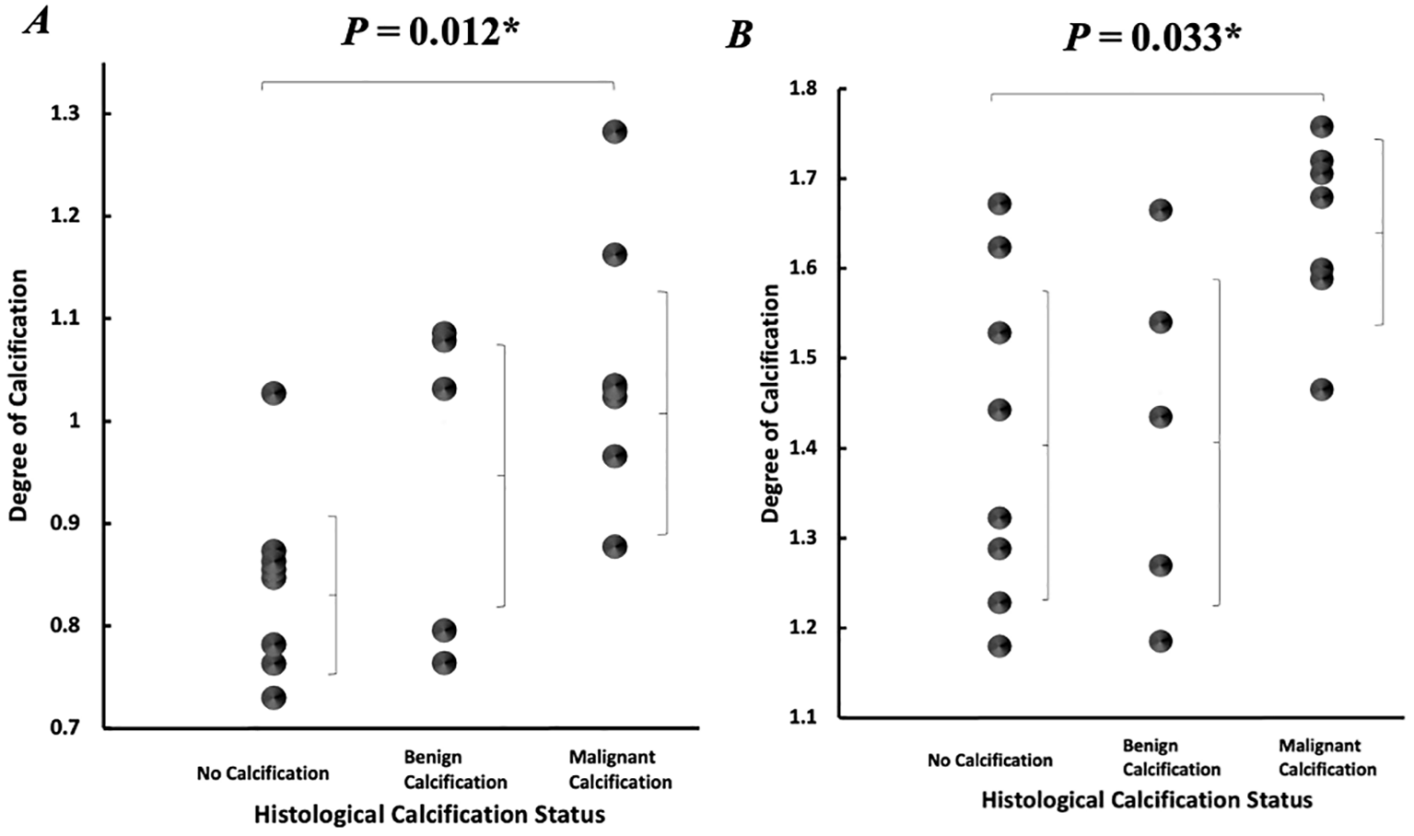
Degree of calcification according to histological calcification status. Individual dots show quantification from each tumour, and error bars indicate mean (standard deviation). **(A)** Intra-tumoural degree of calcification. **(B)** Peri-tumoural degree of calcification. Statistical significance is marked by “*”.

**Figure 4:**
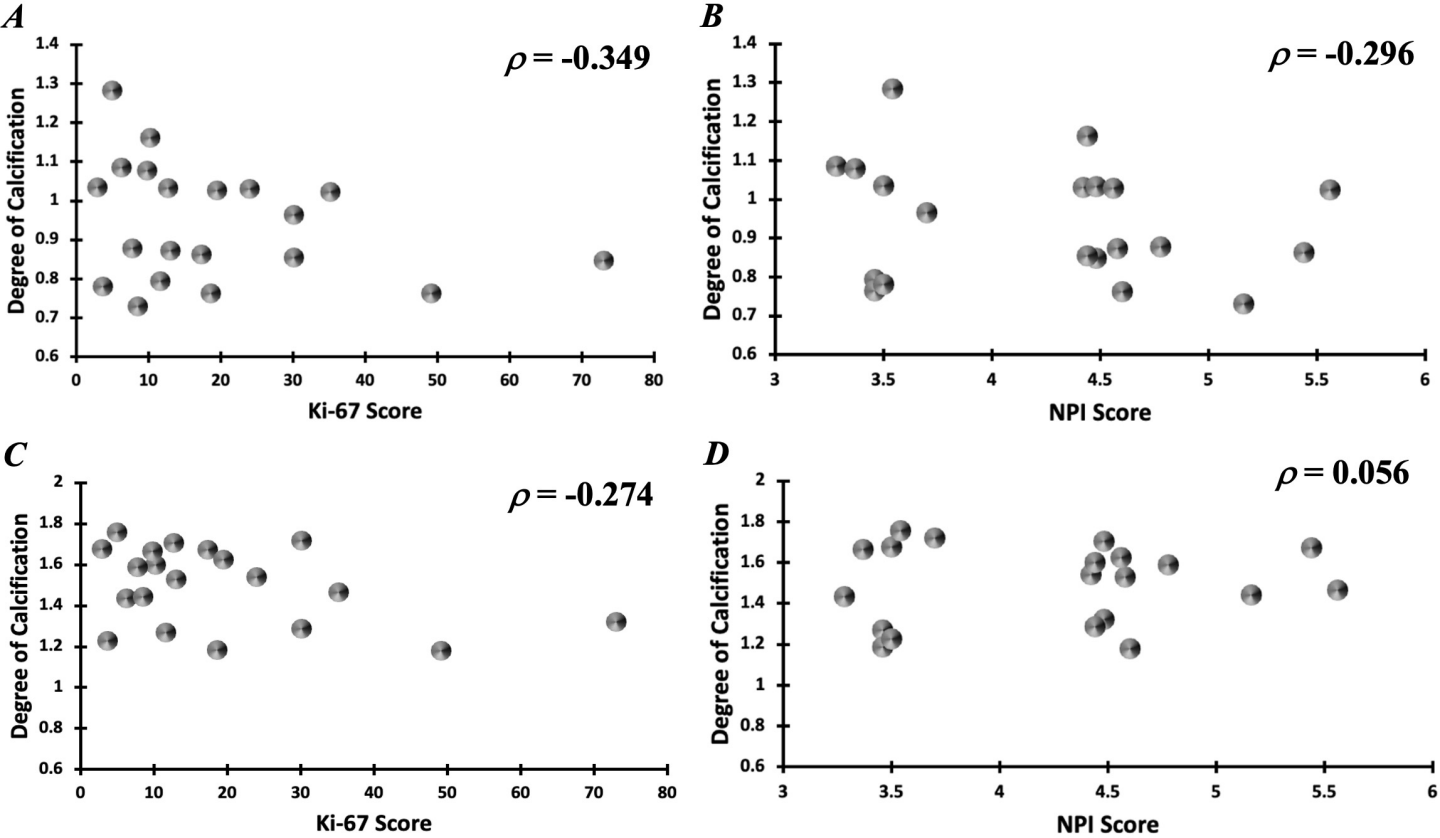
Correlation of intra-tumoural degree of calcification against **(A)** Ki-67 and **(B)** NPI scores. Correlation of peri-tumoural degree of calcification against **(C)** Ki-67 and **(D)** NPI scores.

In the intra-tumoural region, the degree of calcification in malignant classification (1.05 ± 0.13) was significantly higher (p = 0.012) in comparison to no calcification classification (0.84 ± 0.09) (**Figure 3A**). There was no significant difference in the degree of calcification between benign classification (0.95 ± 0.16) and no calcification classification. In the peri-tumoural region, the degree of calcification in malignant classification (1.64 ± 0.10) was significantly higher (p = 0.033) in comparison to no calcification classification (1.41 ± 0.18) (**Figure 3B**). There was no significant difference in the degree of calcification between benign classification (1.42 ± 0.20) and no calcification classification. Neither intra- nor peri-tumoural degree of calcification showed significant correlation against NPI or Ki-67 score (**Figure 4**).

**Figure 5:**
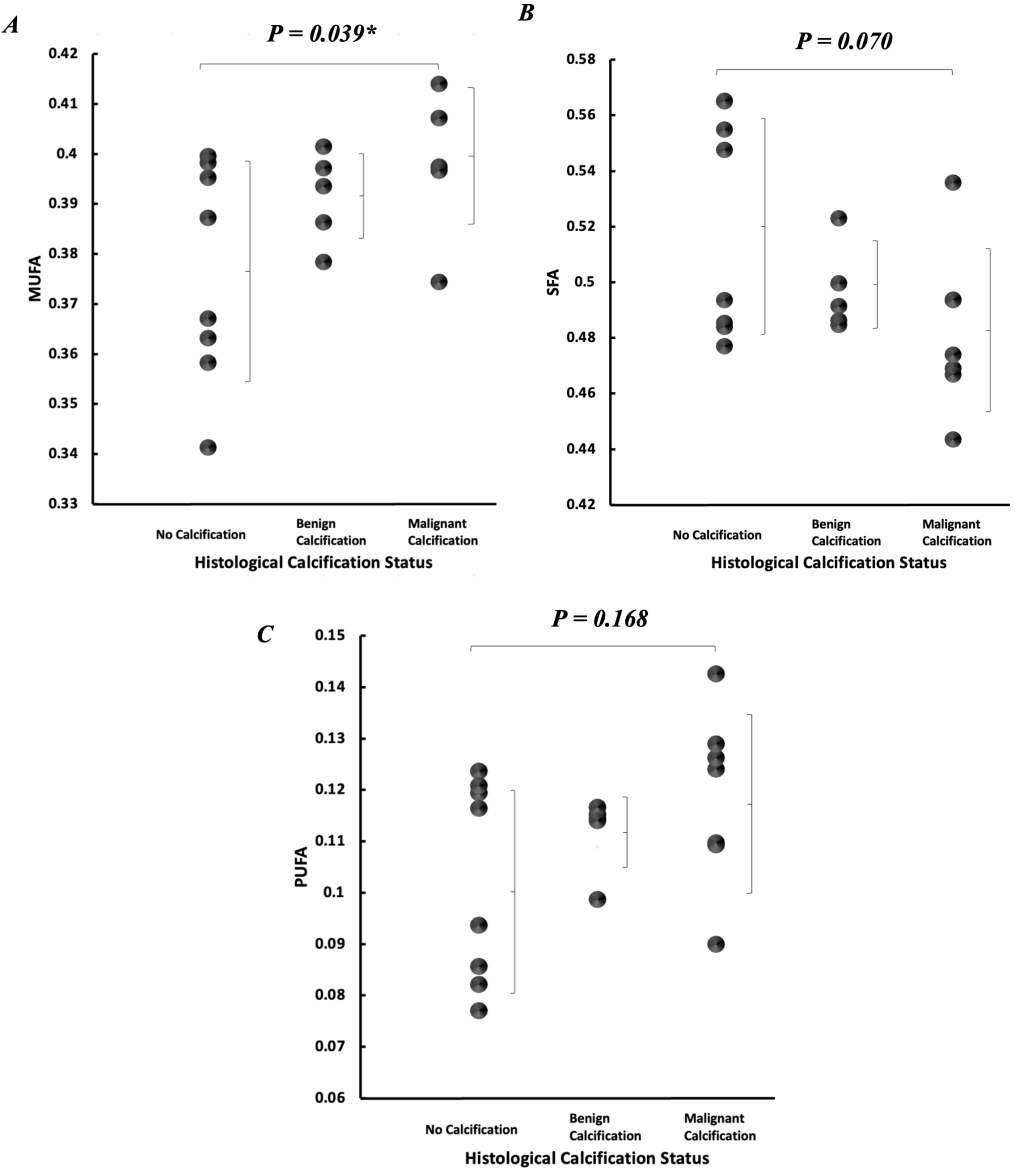
Peri-tumoural lipid composition according to histological calcification status. Individual dots show quantification from each tumour, and error bars indicate mean (standard deviation). **(A)** MUFA, **(B)** SFA, and **(C)** PUFA. Statistical significance is marked by “*”.

**Figure 6:**
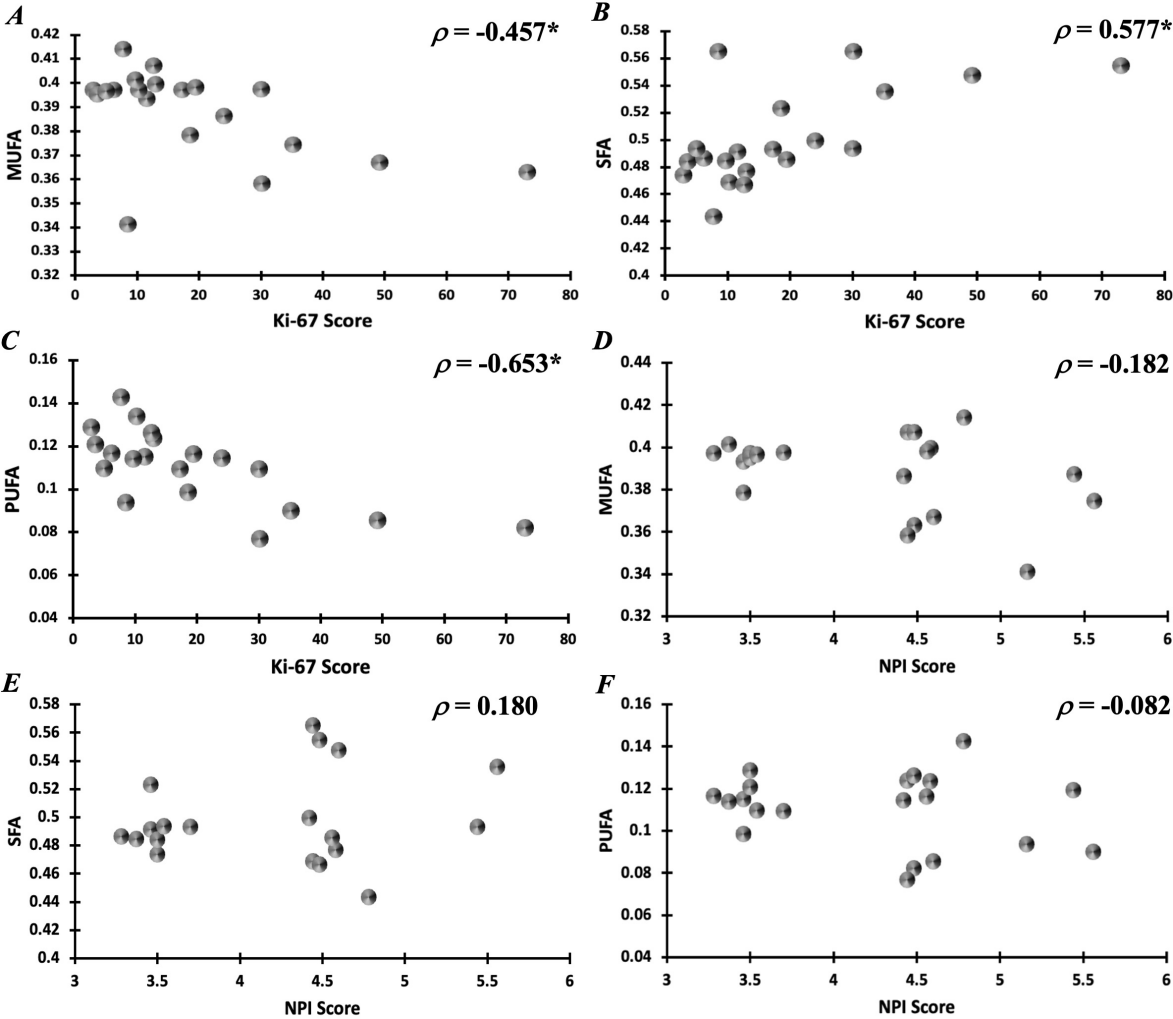
Correlation of peri-tumoural lipid composition of **(A)** MUFA, **(B)** SFA, and **(C)** PUFA against Ki-67 scores. Correlation of peri-tumoural lipid composition of **(D)** MUFA, **(E)** SFA, and **(F)** PUFA against NPI scores. Statistical significance is marked by “*”.

Peri-tumoural MUFA in malignant classification (0.40 ± 0.01) was significantly higher (p = 0.039) in comparison to no calcification classification (0.38 ± 0.02) (**Figure 5A**). There was no significant difference in peri-tumoural MUFA between benign classification (0.39 ± 0.01) and no calcification classification. Peri-tumoural SFA in neither malignant (0.48 ± 0.03) nor benign (0.50 ± 0.02) classifications showed significant difference against no calcification classification (0.52 ± 0.04) (**Figure 5B**). Peri-tumoural PUFA in neither malignant (0.12 ± 0.02) nor benign (0.11 ± 0.01) classifications showed significant difference against no calcification classification (0.10 ± 0.02) (**Figure 5C**). Ki-67 showed significant negative correlation against peri-tumoural MUFA (p = 0.043, ρ = −0.457, **Figure 6A**), significant positive correlation against SFA (p = 0.008, ρ = 0.577, **Figure 6B**), and significant negative correlation against PUFA (p = 0.002, ρ = −0.653, **Figure 6C**). NPI showed no significant correlation against peri-tumoural MUFA (p = 0.442, ρ = −0.182, **Figure 6D**), SFA (p = 0.448, ρ = 0.180, **Figure 6E**), and PUFA (p = 0.731, ρ = −0.082, **Figure 6F**).

## 4 Discussion

We found that the intra- and peri-tumoural degree of calcification and peri-tumoural MUFA were significantly higher in tumours with malignant calcification against tumours with no calcification. Ki-67 was negatively correlated against peri-tumoural MUFA and PUFA, although positively correlated against SFA.

The elevated intra-tumoural degree of calcification in malignant classification against non-calcified classification indicated a higher quantity of 1H nuclei in solid form within the tumour (13, 43, 44), while malignant calcification takes the form of hydroxyapatite (HAP) with higher 1H abundance and is typically more widespread morphologically (13, 43). The presence of calcification is associated with elevated uptake of nutrients by proliferative tumour cells depriving nutrients from intra-tumoural region resulting in the formation of necrotic cores and loss of liquid in surface region (45). Extracellular matrix (ECM) collagens are also associated with the formation of calcification enabling the release of calcium and providing a scaffold for HAP–collagen binding (46). The elevated peri-tumoural degree of calcification in malignant classification against non-calcified classification indicated a higher quantity of 1H nuclei in solid form around the tumour, while the encroachment of basement membrane by tumour cells is a central characteristic of malignant transformation (47). The peri-tumoural calcification may highlight the proliferation of carcinoma cells, typically advancing either retrogradely within the lobule or anterogradely within the ducts, forming linear branching patterns and extend through the breast stroma (48). The degree of calcification in benign classification numerically situates between malignant classification and non-calcified classification without reaching statistical significance for differentiation. The tumours with benign calcification classification may contain both subtypes of calcification (13) leading to a reduction in the quantity of 1H nuclei within solid form compared to HAP alone (43), thus the mild elevation of the degree of calcification. Benign calcification typically assumes a diffuse spatial pattern, instead of segmental, linear, and clustered distribution in malignant calcification (49, 50), which may intrinsically be associated with a lower quantity of calcification, and, in turn, a mild elevation of the degree of calcification (50). Hence, intra- and peri-tumoural calcification might be a marker of malignant calcification supporting future investigation into DCIS prognosis.

The elevated peri-tumoural MUFA in malignant classification against non-calcified classification indicated a higher concentration of MUFA around the tumour in malignant classification, while *de novo* synthesis accelerates the biosynthesis of unsaturated fatty acids (27, 51) and, in particular, MUFA for the vascular calcification in the immediate vicinity around the tumour (52). The negative correlation between MUFA and Ki-67 indicates a MUFA and calcium-enriched micro-environment might be endemic in tumours at early phase of development (27, 51), while the positive correlation between SFA and Ki-67 might be the result of elevated *de novo* synthesis and subsequent increased trafficking of SFA out of a more proliferative tumour to reduce lipotoxicity (36, 53). SFA accumulation has been shown to decrease progressively from the tumour boundary, leading to higher peri-tumoural SFA at close vicinity around the tumour in comparison to wider peri-tumoural region and adipose tissue distal to the tumour (36, 53). The negative correlation between PUFA and Ki-67 was potentially due to an increased utilisation of PUFA in support of local inflammation and elevated membrane synthesis in a more aggressive tumour (53). Our findings are in agreement with literature on benign and invasive breast tissue in invasive ductal carcinoma patients (27) demonstrating the potential of lipid composition for histological calcification differentiation supporting future investigation into DCIS prognosis.

Mammography remains the primary method for identifying DCIS due to its sensitivity to microcalcifications. However, its reliance on spatial distribution and morphological characteristics without molecular specificity often leads to high false-positive rates and a low positive predictive value (PPV) contributing to unnecessary biopsies and overtreatment (9, 10). Standard MRI may detect DCIS; however, a wide variation in sensitivity to calcified and non-calcified tumours is shown (54). Diffusion weighted imaging may reveal a difference in apparent diffusion coefficients between DCIS and IDC, but suffers from low signal-to-noise ratio and incomplete fat saturation. Quantitative DCE MRI shows differential vascular permeability between pure DCIS and DCIS with an invasive component (54, 55), but suffers from low reproducibility (21). PET/MRI, combining functional metabolic imaging with structural MRI, offers greater specificity and the potential for benign and malignant tumour differentiation (56); however, the reliance on radiotracers introduces ionising radiation unsuitable for routine screening applications in early-stage disease. UTE MRI captures rapidly decaying signals from solid-state structures, with the potential of detection and classification of calcification to support DCIS differentiation, in contrast to a lack of strong correlation between mammographic findings and histological results in the differentiation between benign and malignant calcifications. CSEI enables accurate quantification of lipid constituents and is highly sensitive to early pathological changes in fatty acid synthesis offering a potential valuable imaging biomarker for the characterisation and risk stratification of DCIS.

To our knowledge, our study is the first investigation on histological calcification differentiation using UTE and CSEI for calcification and lipid composition. The study was performed on freshly excised whole tumour to avoid the impact of biological noise for the initial demonstration of clinical utility; however, future patient studies are critical for the clinical translation of the methods. The sample size was limited as a proof-of-concept study to highlight the clinical relevance of identifying malignant calcifications as an initial step for understanding DCIS prognosis, with a focus on detection and differentiation of malignant calcification. Future work will address limitations through multicentre studies with larger cohorts to improve statistical power and enable precise stratification by calcification type to achieve the ultimate aim in improving early detection and differentiation of DCIS. The efficacy of UTE MRI should be compared against mammography for specificity in health technology assessment, with pathology as the gold standard. X-ray diffraction studies can be employed to further characterise the biochemical composition of calcifications providing a more robust understanding of their clinical significance. *In vivo* studies are essential for validating the clinical applicability of the imaging biomarkers to bridge the gap between preclinical findings and clinical outcomes. Longitudinal studies tracking calcification and lipid composition changes in relation to disease progression and treatment response will further inform the biological and prognostic significance of these markers.

*Ex vivo* imaging minimises biological noise for high precision, but does not fully replicate the *in vivo* environment, and *in vivo* imaging is required to investigate the dynamic interactions between the tumour and surrounding tissues, including vascularisation (18), formation of calcification (57), and lipid trafficking (36). Our *ex vivo* approach serves as a solid step to establish the utility of UTE MRI and CSEI in breast cancer imaging, and future studies should focus on clinical applications in the patient population. The calcification classification is categorical at an individual patient basis without information on spatial distribution allowing a direct clinical link with pathological reports; however, future spatially resolved investigation using ultra high field MRI is central in the understanding of underpinning mechanism of the imaging marker. The dual-echo approach in this study was selected to optimise the detection of calcifications using UTE MRI, with the shortest achievable TE at 0.17 ms chosen to capture signals from tissues with rapid signal decay, and second TE at 4.60 ms to coincide with water and fat in-phase. Although multi-echo acquisition is technically feasible, and we have conducted testings, the shortest second TE at 0.6 ms is greater than T2 of calcification to detect meaningful signal dynamics from solid matter (58). Based on the T2 relaxation of liquid matter, it is feasible to compute the pixel-wise T2 and the liquid signal at 0.17 ms. However, it is well known that it may introduce errors or noise due to the fitting process (59). Since the short delay between excitation and acquisition in UTE demands the field gradient be switched on at the excitation, the alteration of the first TE does not alter the image contrast to provide more insight into the nature of calcification, but simply leaving a wider area near the centre of k-space leading to blurred images as we have observed during experimental setup (60). In addition, blurring is aggravated at longer TEs due to the accumulation of linear phase errors from gradient imperfections (37).

The mammography images and gradings were not available in the study limiting the analysis purely based on histological findings; future studies co-localising mammography and MRI would allow a direct connection between underpinning pathophysiology and current radiological approach to extract new complementary diagnostic information. Menopausal status of the patients was not collected in this study. Menopausal status is known to impact breast tissue composition contributing to the variations in lipid content and breast density. Postmenopausal women are associated with reduced breast tissue density (61), altered lipid metabolism in the breast (52), and in the presence of microcalcification clusters, an increased risk of breast cancer (62). Future studies should include the collection of menopausal status and analysis on correlation with the degree of calcification and lipid composition to provide a more comprehensive understanding of the metabolic impact and improve the interpretation of the results.

The study highlights the potential of UTE MRI and CSEI as novel imaging methods for differentiating calcification subtypes through calcification load and lipid composition (36) in breast cancer, with the potential to improve the specificity in the diagnosis of DCIS (13). Accurate differentiation of benign and malignant calcifications in DCIS has significant clinical implications, with the potential to optimise treatment and improve patient outcomes (57). Malignant microcalcifications are often the only findings in mammography in women with DCIS before surgery, and residual microcalcifications in the breast indicate incomplete excision significantly increasing the local recurrent rates (63). The correct demarcation of malignant calcification from 3D UTE and CSEI images, acquired without breast compression, might better inform surgical planning in comparison to 2D mammography images, acquired with breast compression, currently contributing to insufficient correlation against pathological findings (13). The improvement in specificity would support the safe adoption of breast conservation and improve guidance for radiotherapy, since breast conservation is not performed in patients with large areas of microcalcifications, and radiotherapy is only recommended for high-grade DCIS avoiding potential overtreatment in low- to intermediate-grade DCIS and undertreatment in high-grade DCIS. Further, microcalcifications persist after neoadjuvant chemotherapy in patients with locally advanced breast cancer, with disagreement between pathological and mammographic response and overestimation of the extent of malignancy in 40% of patients (64, 65). Therefore, the novel imaging methods have the potential to serve as specific treatment monitoring markers for personalised treatment and impacting on patient care pathway.

## 5 Conclusion

In conclusion, the intra- and peri-tumoural degrees of calcification and peri-tumoural MUFA are sensitive to histological calcification classes supporting future investigation into DCIS prognosis.

## Data Availability

The raw data supporting the conclusions of this article will be made available by the authors, without undue reservation.
