# Supplementary material for: Differentiation of histological calcification classifications in breast cancer using ultrashort echo time and chemical shift-encoded imaging MRI
Source: Front Oncol. 2024 Dec 17;14:1475090. doi: 10.3389/fonc.2024.1475090 (PMC11685069; doi:10.3389/fonc.2024.1475090)
Supplement: Supplementary file 1 [file DataSheet1.pdf]

[Supplementary Materials:]

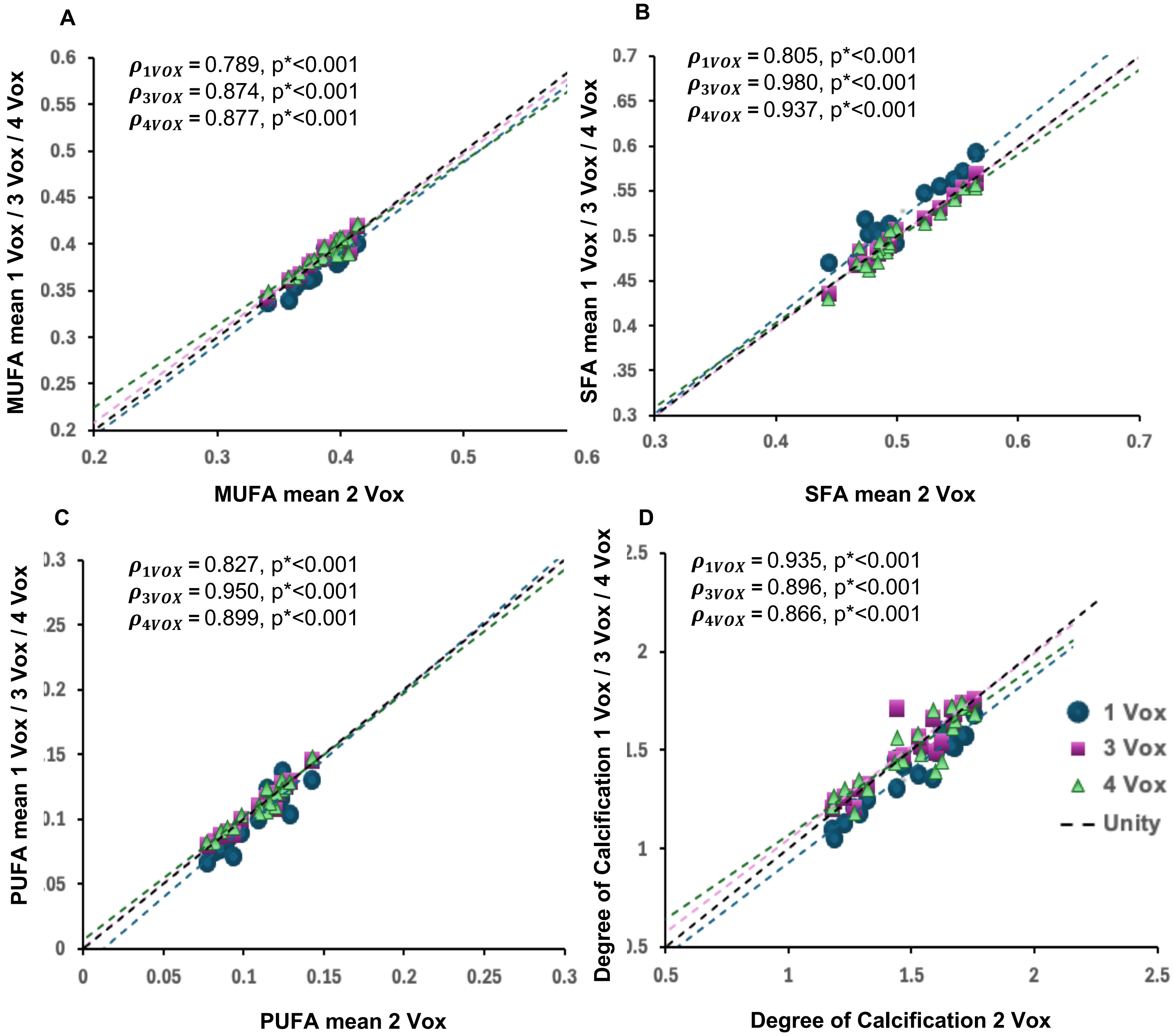

Figure S1 Correlation of peri-tumoural lipid composition and degree of calcification determined at 2 voxels against 1, 3 and 4 voxels. Spearman's rank correlation test was conducted for mean A) MUFA, B) SFA C) PUFA and D) Degree of calcification. All values of *rho* (ρ) were greater than 0.6, with significant correlations across all the metrics.

Table S1. Peri-tumoural degree of calcification and monounsaturated, polyunsaturated and saturated fatty acids (MUFA, PUFA, SFA) at 1, 2, 3 and 4 voxels

The mean degree of calcification, MUFA, PUFA and SFA compared between peri-tumoural regions of 1, 2, 3 and 4 voxels. The paired sample *t*-test was conducted between the threshold choice of 2 voxels (4.4mm, main text) against 1, 3 and 4 voxels. Statistical significant differences ($p < 0.012$) are marked in bold.

	Threshold choice				2 vs 1 voxel	2 vs 3 voxels	2 vs 4 voxels
	1 voxel	2 voxels	3 voxels	4 voxels	*p*	*p*	*p*
MUFA	0.38 ± 0.02	0.39 ± 0.02	0.39 ± 0.02	0.39 ± 0.02	**<0.001**	0.225	0.157
PUFA	0.10 ± 0.02	0.11 ± 0.02	0.11 ± 0.02	0.11 ± 0.02	**0.004**	0.973	0.323
SFA	0.52 ± 0.04	0.50 ± 0.03	0.50 ± 0.04	0.50 ± 0.03	**<0.001**	0.284	0.134
Degree of Calcification	1.39 ± 0.19	1.49 ± 0.19	1.51 ± 0.19	1.49 ± 0.18	**<0.001**	0.353	0.788
